# Sodium Butyrate: A Multifaceted Modulator in Colorectal Cancer Therapy

**DOI:** 10.3390/medicina61010136

**Published:** 2025-01-15

**Authors:** Alexandra Laura Mederle, Alexandra Semenescu, George Andrei Drăghici, Cristina Adriana Dehelean, Nicolae-Valentin Vlăduț, Dragoş Vasile Nica

**Affiliations:** 1 Doctoral School, “Victor Babeș” University of Medicine and Pharmacy Timişoara, Eftimie Murgu Square No. 2, 300041 Timișoara, Romania; alexandra.mederle@umft.ro; 2 Faculty of Pharmacy, “Victor Babeș” University of Medicine and Pharmacy Timișoara, Eftimie Murgu Square No. 2, 300041 Timișoara, Romania; alexandra.scurtu@umft.ro (A.S.); cadehelean@umft.ro (C.A.D.); 3 Research Center for Pharmaco-Toxicological Evaluations, Faculty of Pharmacy,“Victor Babeş” University of Medicine and Pharmacy Timișoara, Eftimie Murgu Square No. 2, 300041 Timișoara, Romania; nicadragos@gmail.com; 4 The National Institute of Research—Development for Machines and Installations Designed for Agriculture and Food Industry (INMA), Bulevardul Ion Ionescu de la Brad 6, 077190 București, Romania; vladut@inma.ro

**Keywords:** sodium butyrate, colorectal cancer, CRC, apoptosis

## Abstract

*Background and Objectives*: Sodium butyrate (NaB) is a potent modulator of cancer-related gene networks. However, its precise mechanisms of action and effects at elevated doses remain insufficiently explored. This study investigated the impact of NaB at physiologically relevant doses on key cellular metrics (viability, confluence, cell number, morphology, nuclear integrity) and a comprehensive set of apoptosis and proliferation regulators (including underexplored genes) in colorectal cancer (CRC) cells. *Materials and Methods*: Human HCT-116 cells were treated with increasing NaB concentrations (0–20 mM). Cell viability, confluence, number, morphology, and nuclear integrity were assessed using MTT and imaging assays. RT-PCR was used to determine changes in the expression of critical pro-apoptotic players (*BAX*, *CASP3*, *PUMA*, *TP53*), anti-apoptotic facilitators (*BCL*-2, *MCL-1*), cell division regulators (*PCNA*, *Ki-67*, *CDKN*1), and inflammation genes (*NF-κB*). *Results:* This study provides the first exploration of *MCL-1* and *PCNA* modulation by NaB in the context of CRC and HCT-116 cells, offering significant translational insights. All treatments reduced cell viability, confluence, and number in a dose-dependent manner (*p* < 0.0001). Gene expression revealed dose-related increases in most pro-apoptotic markers (*BAX*, *CASP3*, *PUMA*; *p* < 0.001), and decreases for the other genes (*p* < 0.001). BAX emerged as the most responsive gene to NaB, while TP53 showed minimal sensitivity, supporting NaB’s effectiveness in p53-compromised phenotypes. Nuclear condensation and fragmentation at higher NaB doses confirmed apoptotic induction. *Conclusions*: NaB can modulate critical apoptotic and cell cycle genes, disrupt tumor cell proliferation, and overcome resistance mechanisms associated with anti-apoptotic regulators such as *MCL-1*. By targeting both short-term and long-term anti-apoptotic defenses, NaB shows promise as a preventive and therapeutic agent in CRC, particularly in high-risk phenotypes with compromised p53 functionality. These findings support its potential for integration into combination therapies or dietary interventions aimed at enhancing colonic butyrate levels.

## 1. Introduction

Together with acetic acid and propionic acid, butyric acid (C_4_H_8_O_2_) is one of the three main short-chain fatty acids (SCFAs) resulting from the intestinal fermentation of dietary fibers by gut microbiota [1,2]. Despite accounting for only 10–15% of the total SCFAs in the gut [3], it exerts the greatest physiological effects [4]. Having resistant starches and inulin as the primary substrates, this microbial metabolite is essential for preserving the integrity of the intestinal barrier [5]. Butyrate-producing bacteria hence control the access and proliferation of other bacteria, especially pathogenic types, within the gut [4,5]. This fatty acid also serves as the primary energy source for colonocytes, i.e., the cells lining the colon [6,7]. Moreover, it displays a broad spectrum of beneficial features; among others, anti-inflammatory and anti-cancer properties—especially against colorectal cancer (CRC) [8,9]. This type of cancer remains the third most commonly diagnosed cancer type and the second cause of cancer-related mortality worldwide despite recent advances in its prevention and treatment [10,11].

Strong evidence indicates that butyrate derived from dietary fibers provides protection against colorectal cancer in a dose-dependent manner [12]. Among different salts of butyric acid, sodium butyrate (NaB) is widely used in CRC research due to its stability, bioavailability, safety, and well-documented biological effects on key molecular pathways in cancer [9]. This compound is a potent modulator of gene expression. Working as a histone deacetylase (HDAC) inhibitor, NaB promotes a more disordered/open chromatin structure, thus facilitating increased accessibility of transcriptional machinery to DNA [9,13]. A recent review by Kaźmierczak-Siedlecka et al. (2022) shows that it exerts multiple anti-carcinogenic effects against different CRC lines. Thus, this butyrate salt induces cell cycle arrest and apoptosis in cancer cells without affecting normal cells, with over 7000 genes being differentially expressed in treated cells. NaB promotes autophagy via the liver kinase B1 (*LKB1*) and AMP-activated protein kinase (*AMPK*) pathway (i.e., *LKB1*/*AMPK* signaling pathway). This compound also inhibits CRC cell migration via enhancement of miR-200c expression-mediated downregulation of B lymphoma Mo-MLV insertion region 1 (Bmi-1)—a pivotal protein in modulating chromatin structure. Moreover, NaB enhances the expression of several proteins encoded by the cyclin-dependent kinase inhibitor 2A/2B (*CDKN2A/2B*) gene, such as p16INK4a, p14ARF, and p15INK4b, which serve as critical regulators of cell cycle control and defense against tumorigenesis [9]. Importantly, it modulates the *Wnt* signaling, nuclear factor kappa B (*NF-κB*), and the p53 pathways—all of which are critical for the maintenance of cellular homeostasis and the progression of cancer [14]. We emphasize its ability to induce cell proliferation blockade in the G1 phase and promote apoptosis via upregulation of both pro-apoptotic genes (e.g., *BAX*, *PUMA*) and downregulation of anti-apoptotic genes (e.g., *BCL-2*) [9,15]. Consequently, it has been attracting growing research interest in the context of cancer, and especially CRC [16,17,18]. However, its precise mechanism of action is still far from being fully elucidated [9,14,18].

The human CRC cell line HCT-116 is routinely used—both in two-dimensional and three-dimensional culture models—to investigate cellular interactions, drug efficacy, and tumor microenvironment dynamics [18]. Most studies to date have investigated the role of NaB in modulating apoptosis and cell cycle arrest, focusing on well-characterized genes such as *BAX*, *BCL-2*, *CASP3*, and *TP53* [14,19,20,21,22,23,24,25]. These markers are very important for understanding how this compound influences apoptosis pathways and tumor suppressor functions [24]. Another frequently examined gene is *CDKN1A* (p21), which relates to cell cycle regulation [24]. In contrast, less information is available for *Ki-67* (*MKI67*), a marker for proliferation in CRC studies [24]. There is also a paucity of data for other genes, e.g., *PCNA* (Proliferating Cell Nuclear Antigen) or *MCL-1* (Myeloid Cell Leukemia 1), despite their potential importance in understanding CRC. More precisely, *PCNA* is often analyzed as a marker for cell proliferation, but its role in CRC remains somewhat controversial as a prognostic factor [26]. *MCL-1*, an anti-apoptotic protein, contributes to chemoresistance [27], and targeting it may help in overcoming resistance in CRC treatments. In addition, the bulk of the data on NaB and CRC derives from experiments with doses ranging from 0.5 millimolar (mM) to 10 mM. Moreover, the impact of higher doses, such as 15 mM and 20 mM, on cancer cell survival, proliferation, and apoptosis, especially in more resistant cancer phenotypes, remains unclear.

The current study aimed to expand the current knowledge of gene networks underlying the anti-cancerous effect of NaB in the context of CRC. In this context, we have analyzed changes in the expression of a comprehensive set of key genetic actors in apoptosis and cell proliferation. This set of genes includes (*i*) apoptosis (cell death) regulators, that is *BAX* (BCL2 Associated X, Apoptosis Regulator): pro-apoptotic, promoting cell death; *BCL-2* (B-Cell Lymphoma 2): anti-apoptotic, preventing cell death; *CASP3* (Caspase 3): executioner caspase in apoptosis; *PUMA* (BBC3, BCL2 Binding Component 3): pro-apoptotic, inducing cell death; *TP53* (Tumor Protein P53): inducer of apoptosis in response to cellular stress; *MCL-1* (Myeloid Cell Leukemia 1): anti-apoptotic, aiding in cell survival; and *NF-κB* (NFKB1, Nuclear Factor Kappa B Subunit 1): regulator of genes for cell survival and inflammation; and (*ii*) genes involved in cell division and proliferation, more precisely *PCNA* (Proliferating Cell Nuclear Antigen): marker of cell proliferation; *Ki-67* (MKI67, Marker of Proliferation Ki-67): indicator of cell growth; and *CDKN1A* (p21, Cyclin Dependent Kinase Inhibitor 1A): regulator of cell cycle arrest. We also determined the effect of NaB on the viability, confluence, cell number, and morphology of HCT-116 colorectal carcinoma cells. In contrast to prior investigations that concentrated on lower concentrations or single molecular pathways, our work examined multiple apoptotic and proliferative genes (including less studied markers, e.g., *MCL-1*, *PCNA*) over a broad range of physiologically relevant butyrate levels (5, 10, 15, and 20 mM). Refining the current understanding of how NaB influences these critical genes could guide future drug development and treatment strategies. Moreover, our findings uncover new aspects of the complex interplay between NaB and apoptosis, thus offering new insights into its multifaceted role in CRC prevention and treatment.

## 2. Materials and Methods

### 2.1. Cell Culture Conditions

McCoy’s 5A medium, the mixture of penicillin/streptomycin (pen/strep), and the trypsin-EDTA solution were acquired from ATCC (American Type Culture Collection, Manassas, VA, USA). Fetal bovine serum (FBS) and the MTT (3-(4,5-dimethylthiazol 2-yl)-2,5-diphenyltetrazolium bromide) kit were purchased from Sigma Aldrich (Steinheim, Germany). The lactate dehydrogenase (LDH) kit and the Hoechst 33342 dye were provided by Thermo Fisher Scientific Inc. (Waltham, MA, USA). The human colorectal carcinoma cell line HCT-116 was obtained from ATCC as a frozen vial. The cells were grown in their specific culture medium supplemented with 1% antibiotic mixture and 10% FBS. The cells were maintained under controlled laboratory conditions in a humidified incubator at 5% CO_2_ and a temperature of 37 °C.

### 2.2. Viability Analysis

Cell viability was determined with the MTT method. Briefly, HCT-116 cells were grown in 960-well culture plates (104 cells/well) and exposed for 24 h to different concentrations of NaB diluted in H_2_0 (1–20%). After this time period, the medium was refreshed with a new solution—100 microliters (µL) per well. In each well, 10 µL of 1 MTT solution was next added, and the plate was incubated for 3 h. The resulting formazan crystals were dissolved in 100 µL/well of solubilization buffer (30 min. in the dark). Finally, the amount of reduced MTT was measured spectrophotometrically at a wavelength of 570 nanometers (nm) with a Cytation 5 microplate reader (BioTek Instruments Inc., Winooski, VT, USA) [28,29].

### 2.3. Cell Confluence and Cell Number Evaluation

Images were acquired in high-contrast bright-field mode at four-fold (4×) magnification using a Biotek Lionheart™ FX automated microscope optimized for live imaging (Agilent, Santa Clara, CA, USA). Each photo was analyzed using the Cell Analysis Tool provided by the Gen5 Microplate Data Collection and Analysis Software (Version 3.14).

### 2.4. Assessment of Cellular Morphology

To evaluate the changes induced by NaB in terms of morphology, the HCT-116 cells were microscopically examined under bright-field illumination at a magnification level of ×20. The images were acquired as described above (see Section 2.3).

### 2.5. Nuclear Morphology Evaluation

The Hoechst nuclear staining assay was applied to define the type of cell death induced by NaB [27]. In short, cells were grown in 12-well plates (1 × 10^5^ cells/well) and treated with NaB at different concentrations when reaching approximately 80–90% confluence. At the end of the experiment, the medium was removed, and 500 μL of Hoechst staining solution/well (1:2000 in PBS) was added. After incubation for 10 min at room temperature and with protection from light, the Hoechst solution was removed and washed 3× with PBS. The images were processed as described above (see Section 2.3).

### 2.6. Gene Expression

RNA was extracted using the Quick-RNA Miniprep Kit (Zymo Research, Irvine, CA, USA) as per the manufacturer’s protocol. The concentration and purity of the extracted RNA were determined using a DS-11 spectrophotometer (DeNovix, Wilmington, DE, USA). Reverse transcription was run with the Maxima^®^ First Strand cDNA Synthesis Kit (Thermo Fisher Scientific, Inc., Waltham, MA, USA) in accordance with the manufacturer’s guidelines [29]. Quantitative real-time PCR (RT-qPCR) was next performed using the Quant Studio 5 real-time PCR system (Thermo Fisher Scientific, Inc., Waltham, MA, USA), employing the Power SYBR- Green PCR Master Mix (Thermo Fisher Scientific, Inc., Waltham, MA, USA) for the amplification of specific gene sequences [30]. Primer sequences specific to *BAX*, *BCL-2*, *PCNA*, *Ki-67* (*MKI67*), *CASP3*, *PUMA* (*BBC3*), *TP53*, *MCL-1*, *NF-κB* (*NFKB1*), and *CDKN1A* (*p21*) genes were used to ensure targeted amplification. Expression data sets were normalized against the housekeeping gene *18S*. The primer oligonucleotides utilized in this study were provided by Thermo Fisher Scientific, Inc. (Waltham, MA, USA). Their sequences are presented in Table 1.

### 2.7. Statistical Analysis

Prior to applying one-way ANOVA, data sets were verified for normality and homogeneity of variances (homoskedasticity) using Shapiro–Wilk tests and Levene’s tests, respectively [31,32]. In case of significant results for ANOVA, post hoc testing was run against controls using Dunnet’s tests [33]. Employing a minimum of six concentrations, spaced logarithmically, is recommended for accurate IC_50_ determination. For a smaller number of concentrations and exploratory investigations, which was the case of our study, the four-parameter logistic regression (4PL) is a viable alternative for obtaining an indicative IC_50_ [34]. Statistical significance was set at a *p*-value threshold of 0.05 [35], and data were analyzed with the GraphPad Prism version 9.4.0 for Windows (GraphPad Software Inc., San Diego, CA, USA).

## 3. Results

### 3.1. NaB Exerts Cytotoxic Effects on the CRC Cell Line HCT-116

**Figure 1:**
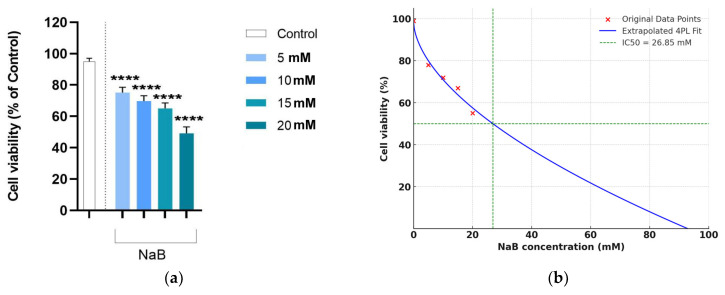
(**a**) The viability of HCT-116 cells at 24 h post-treatment. The data were obtained via the MTT assay and are given as average percentage (normalized to control cells) with one standard deviation. Marked bars (*) indicate significant differences compared to controls (Dunnet’s tests, ****—*p* < 0.0001, ***—*p* < 0.001, **—*p* < 0.01, *—*p* < 0.05). (**b**) The corresponding dose–response curve fitted with the 4PL regression model. The sigmoidal curve indicates a gradual viability reduction, with an indicative IC_50_ of ≈26.85 mM. Error bars represent standard deviations, and the fitted curve reflects the overall trend.

All data sets have met the assumptions of ANOVA, being normally distributed (Shapiro–Wilk test, *p* ≥ 0.262) and homoskedastic (Levene’s test, *p* ≥ 0.154). Mean cell viability values, expressed as percentages of control values with one standard deviation, are presented in Figure 1a. The corresponding dose–response curve fitted using the 4PL regression model is shown in Figure 1b. It was found that NaB induces a significant, dose-dependent decrease in viability in the colon cancer cell line HCT-116 at 24 h (ANOVA, *p* < 0.001). After starting at an initial viability of 81% versus controls, the maximum decrease in viability was observed at the highest treatment dose, being about 49% of the values observed for the reference group (Figure 1a). The dose–response curve fitted using the 4PL regression model yielded an indicative IC_50_ value around 27.0 mM (Figure 1b).

**Figure 2:**
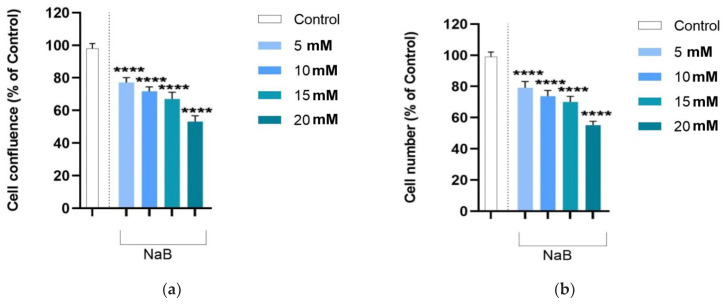
The measured values for (**a**) the cell confluence area and (**b**) the cell number at 24 h post-treatment. The data are given as average percentage (normalized to control cells) with one standard deviation. Marked bars (*) indicate significant differences compared to controls (Dunnet’s tests, ****—*p* < 0.0001, ***—*p* < 0.001, **—*p* < 0.01, *—*p* < 0.05).

Average values with one standard deviation for cell confluence area and cell number (calculated as a percentage of control values) are shown in Figure 2a,b. The same significant, dose-dependent decrease observed for cell viability was found for cell confluence (ANOVA, *p* < 0.001; Figure 2a) and cell number (ANOVA, *p* < 0.001; Figure 2b). We note that the magnitude of decrease at the lowest treatment dose and highest treatment dose was comparable to that seen for cell viability (Figure 1a and Figure 2a,b). For all these parameters, the measured values decreased by almost half after 24 h of exposure to the highest treatment dose.

**Figure 3:**
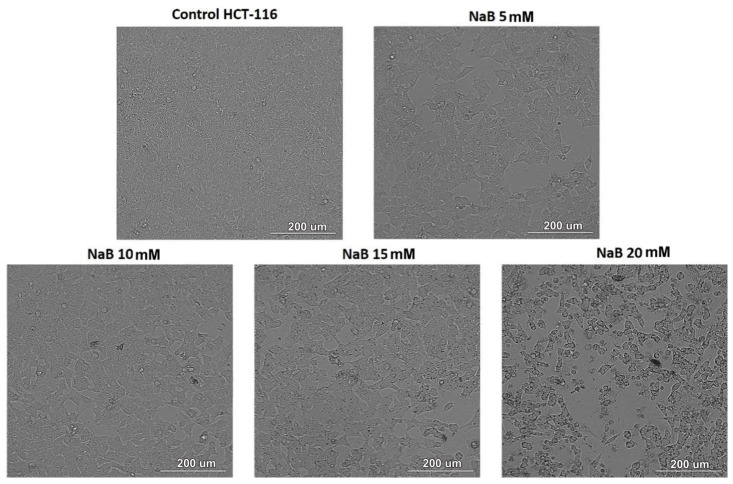
The morphological aspect of the colon cancer cell line HCT-116 after 24 h of treatment with NaB was observed under 20× magnification. Control,cells exhibit (**top left corner**) normal morphology, with intact cell membranes and uniform density across the field of view. At 5 mM (**top right corner**), mild alterations and reduced density were noted. At 10 mM (**bottom left corner**), cell shrinkage and apoptotic bodies became evident. Higher doses of 15 mM (**middle bottom**) and 20 mM (**bottom right corner**) led to severe cell fragmentation, condensed nuclei, and significant density reduction, indicating apoptosis. Scale bars represent 200 µm.

Figure 3 shows a series of microscopic images that compare the effects of different concentrations of NaB on HCT-116 cells at 24 h post-treatment. Cells started to show slight signs of morphological changes at 5 mM, with noticeable changes occurring from a concentration of 10 mM onward (Figure 3). The cell density at this treatment was lower, suggesting reduced viability at this concentration (Figure 3). The density of the cells decreased in a dose-dependent manner for the other treatment groups (Figure 3). This suggests that higher Na B concentrations within the 0–20 mM range may be associated with more severe cytotoxic effects.

**Figure 4:**
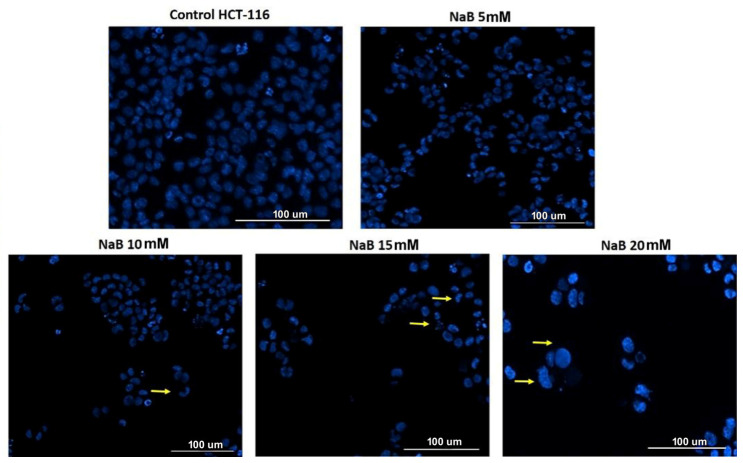
Morphological changes in nuclei after 24 h of NaB treatment were observed under 20× magnification. Control nuclei were intact (**top left corner**), while 10% of cells at 5 mM (**top right corner**) showed mild condensation. At 10 mM (**bottom left corner**), 25% displayed condensation and fragmentation, increasing to about 50% at 15 mM and 70% at 20 mM ((**middle bottom**) and (**bottom right corner**), respectively), where extensive nuclear disintegration was observed. Yellow arrows indicate apoptotic features; scale bars represent 100 µm.

Figure 4 includes a series of fluorescence microscopy images revealing the effects of various NaB concentrations on colon cancer cell line HCT-116 at 24 h post-treatment. In the control group, the nuclei were largely intact and uniform, showing no drastic morphological changes (Figure 4). At the lowest treatment dose (5 mM), about 10% of cells exhibited mild nuclear condensation and the initial stages of chromatin aggregation at the nuclear periphery; this was potentially related to early signs of apoptosis (Figure 4). Noticeable changes started to appear at the second lowest dose (10 mM), i.e., a decrease in cell density and around a quarter of cells having nuclei with clear signs of condensation or fragmentation (Figure 4). These changes become more pronounced with increasing NaB concentrations, with about 50% and 70% of HCT-116 cancer cells revealing signs of nuclear condensation and fragmentation at 15 mM and 20 mM, respectively (Figure 4). At the highest treatment group (20 mM), completely disintegrated nuclei were observed in numerous cells (Figure 4).

### 3.2. NaB Promotes Apoptosis and Triggers Cell Cycle Arrest in HCT-116 Colon Cancer Cells

NaB exerted a significant effect on the expression of analyzed genes (ANOVA; *CASP3*, *PUMA*, and *TP53*, *p* < 0.001; for the other genes, *p* < 0.0001). Table 2 shows NaB-induced changes in gene expression depending on treatment dose. The expression of the pro-apoptotic gene *BAX* increased significantly with NaB dose, starting from the second lowest dose (10 mM) onward (Table 2). The same pattern was observed for the anti-apoptotic markers *BCL-2* and *MCL-1* (Table 2). Comparable results were obtained for *CASP3*, a key executioner of apoptosis, and *PUMA*, an important modulator of apoptosis. In the case of these genes, however, the effect of NaB was evident only from the second-highest dose (15 mM) onward (Table 2).

The expression of *PCNA* and *Ki-67*, well-known cell proliferation indicators, decreased significantly from the second-lowest dose (10 mM) onward, with the former gene being more responsive to NaB treatment (Table 2). The tumor suppressor gene *TP53* showed a moderate but significant reduction in its expression starting at the second-highest dose (15 mM) (Table 2). In fact, it was the gene with the smallest changes in expression. A significant dose-dependent decrease in transcript levels was also observed for the *NF-κB* gene—a key player along the pro-survival signaling pathway—and the *CDKN1A* gene—a major actor in cell cycle arrest. In both these cases, this effect occurred starting from the second-lowest treatment (10 mM). The effect of increasing concentrations of NaB on gene expression changes did not appear to diminish at the highest treatment dose (20 mM), irrespective of the gene examined.

## 4. Discussion

The findings of this study provide valuable insights into the mechanisms of NaB action in colorectal cancer. Its contributions are threefold. First, to our knowledge, this is the first study to demonstrate that *MCL-1*, an anti-apoptotic gene crucial for chemoresistance in colorectal cancer, undergoes dose-dependent downregulation in butyrate-treated CRC cells. Second, it is among the few investigations to establish a direct link between NaB treatment and the modulation of *PCNA*, a key marker of cell proliferation. Finally, it stands out by comprehensively analyzing a wide range of apoptotic markers (e.g., *BAX*, *CASP3*, *PUMA*) and proliferative markers (e.g., *Ki-67*, *PCNA*) across a physiologically relevant dose range of NaB (5–20 mM). Consistent with previous research [6,9,14,16,19,20,21,22,23,24,25], our results render sodium butyrate as a potent agent in both the prevention and treatment of colorectal cancer. The present experiments were conducted at NaB concentrations ranging between 1 and 20 mM, which is in line with physiologically relevant levels. Thus, the proportion of SCFAs in the colonic lumen is 3:1:1 for acetate, propionate, and butyrate, respectively. The highest concentrations are found in the proximal colon, i.e., 7–140 mM [8,16,36]. This corresponds to an upper estimate of butyric acid concentration of 25–30 mM in the colon of healthy individuals.

The expression of most pro-apoptotic markers (i.e., *BAX*, *CASP3*, *PUMA*) increased significantly with treatment dose, with *BAX* emerging as the most responsive gene to NaB action. The role of BAX as an early and central initiator of mitochondrial apoptosis [37], together with its direct responsiveness to the tumor suppressor protein p53 and NaB-induced epigenetic changes [38], may help explain its faster and stronger induction compared to *CASP3* and *PUMA*. The expression of anti-apoptotic genes *BCL-2* and *MCL-1*; cell division regulators *PCNA*, *Ki-67*, and *CDKN1*; and the inflammation modulator *NF-κB*, by contrast, displayed a significant dose-dependent decrease in transcript levels following this treatment. These data underscore the ability of sodium butyrate to simultaneously promote apoptosis and suppress proliferation in HCT-116 cells [6,9,14,16,39].

Interestingly, *TP53* showed the smallest change in expression in response to NaB treatment. It can, therefore, be inferred that sodium butyrate exerts its effects primarily through downstream targets rather than significantly affecting p53 expression levels. Indeed, this compound induces the apoptosis of colon cancer cells in humans via mechanisms independent of the tumor suppressor protein p53 [36]; that is, histone deacetylase inhibition, activation of death receptors, and modulation of signaling pathways [40,41,42]. Of note, about 43% of CRC cases exhibit a loss of tumor suppressor function and resistance against standard treatments due to the presence of TP53 mutations [42,43]. This gene is crucial in the transition from adenoma to adenocarcinoma, marking a critical step in tumor progression [43]. These results favor the use of NaB in the hard-to-treat and high-risk phenotype of colorectal cancer with compromised p53 functionality [43].

Among the genes examined, *Ki-67*, *MCL-1*, and *PCNA* have rarely been the primary focus in studies investigating the effect of sodium butyrate on colorectal cancer cells. A key driver of cell division, the former gene serves as a marker for CRC aggressiveness and is associated with poor overall survival and disease-free survival [44]. As a result, a significant reduction in *Ki*-67 transcript levels indicates a decrease in tumor cell proliferation following NaB treatment. Nonetheless, caution is needed when interpreting these results. For example, the expression of *Ki-67* in 3D colorectal cell models (in vitro cultures) can display at both nuclear and non-nuclear locations, thereby limiting its utility as a proliferation marker in these models [45].

The *MCL-1* gene is crucial in enabling cancer cells to resist apoptosis [46]. Its overexpression is linked to chemoresistance and a poor prognosis in several cancer types, including colorectal cancer [27,47]. The little information available on the effect of NaB on this gene in the context of CRC originates from studies conducted on the human colocarcinoma HT-29 cell line. It was thus found that the administration of polyunsaturated fatty acids enhances NaB-induced apoptosis, resulting, among other changes, in *CASP3* and *MCL-1* downregulation [48]. These data are congruent with our findings. This decrease in *MCL-1* expression is of translational interest, providing evidence for the ability of sodium butyrate to overcome resistance and sensitize CRC cells to apoptosis. Since this compound targets both *BCL-2* and *MCL-1*—important for short-term and long-term anti-apoptotic defenses [46,49], respectively—its dual action supports that it is effective against aggressive and resistant cancer subtypes.

Similar to *MCL-1*, this is the first study to connect PCNA and sodium butyrate in the context of the HCT-116 cell line. This gene is regarded as a biomarker of disrupted cell proliferation in neoplastic epithelial colonic lesions, with the potential to detect high-risk colorectal adenomas with aggressive and malignant potential [50]. Its overexpression contributes to immune evasion by tumors via inhibition of natural killer (NK) cell effector functions [26]. In this study, *PCNA* showed a dose-depedent decrease following NaB treatment, thus aligning with its known anti-proliferative effects [9,16]. Based on these data, it can be inferred that NaB may block the inhibition of NK cell effector functions in CRC by downregulating *PCNA* expression; disrupting its suppressive interaction with NK cell effectors; and reprogramming both the tumor cells and the tumor microenvironment. In this context, it is also important to mention that among SCFAs, only butyrate reduces the invasion of primary human colon cancer cells by reducing *PCNA* expression [51]. This highlights the potential of NaB as an immune-modulatory and anti-proliferative agent in CRC treatment.

Taken together with the aforementioned findings, the gene expression patterns observed here indicate that NaB targets both apoptosis controllers and cell division markers to effectively curb CRC tumorigenicity. On the other hand, one can argue that the downregulation of tumor suppressor genes, such as *TP53* and *CDKN1A*, may lead to a loss of control mechanisms critical for tumor suppression. Robust data challenge this assertion. Thus, a growing body of evidence supports that the p53-mediated DNA damage response is dispensable for tumor suppression since this protein also modulates many other key cellular processes, including metabolism, autophagy, and cell migration. In fact, a reduction in *TP53* expression does not completely shut down tumor suppression mechanisms [52]. Working on three different CRC lines, Encarnação et al. (2019) also demonstrated that butyrate exerts anti-proliferative effects by inhibiting *TP53* and *CDKN1A* activities and increasing the *BAX*/*BCL-2* ratio [53]. These insights indicate that NaB exerts its anti-proliferative and pro-apoptotic effects through a multifaceted approach.

At the cellular level, the anti-cancer effects of NaB became evident starting at concentrations of 5 mM and above, as evidenced by changes in cell viability, proliferation, and morphology. At the gene level, by contrast, this response occurred at higher concentrations. Lower levels of sodium butyrate may, therefore, favor the regulation of subtle processes, such as metabolism and cell cycle control. Higher levels, by contrast, may cause more robust changes, such as apoptosis and cell cycle arrest. This hypothesis is in agreement with the current understanding of the dose-dependent effects of NaB on cellular processes [9,16,18].

The value of IC_50_ according to the 4PL regression model was around 27 mM. Indeed, this regression approach has several drawbacks in fitting dose–reponse curves, such as sensitivity to data quality, overfitting small datasets, and challenges with convergence or applicability to non-sigmoidal data, thus requiring careful data preparation and validation [54]. However, it is recommended in exploratory studies with limited concentrations—as was the case of our study—because it effectively models the underlying biology, minimizes overfitting, and provides meaningful parameters that align well with the experimental data [54]. Notably, this value corresponds to a concentration of 25 mM butyric acid—the upper estimate of levels normally occurring in the colon of healthy individuals. This threshold is consistent with certain previously published results, although not universally in laboratory investigations using the same cell line. For example, Ghiaghi et al. (2019) reported IC_50_ values of 35.5 mM, 9.6 mM, and 10 mM after 24, 48, and 72 h of treatment, respectively, using similar experimental conditions [55]. However, Pattayil et al. (2019) reported lower values, that is, 9.53 mM, 7.83 mM, and 5.98 mM, for the same time points [56]. Li et al. (2024) also identified a lower half-maximal inhibitory concentration at 24 h, i.e., 6.21 mM NaB [57]. In contrast, Mashayekhi et al. (2021) found a higher IC_50_ estimate, namely 50 mM [22]. Variations in experimental conditions (e.g., treatment duration, cell culture protocols) or methodologies (e.g., cell viability measurement techniques, NaB purity) can, at least partly, account for these differences.

Overall, these results indicate that sodium butyrate exerts anti-cancer effects by modulating key molecular pathways in cell proliferation, survival, apoptosis, and death. We note that the effect of increasing NaB concentrations on gene expression did not plateau or diminish at the highest dose tested. One can, hence, advocate for including NaB in dietary strategies, e.g., via fiber-rich diets or targeted probiotic supplementation. This approach, aimed at increasing colonic butyrate concentrations, could offer preventive benefits or serve as an adjuvant to therapy for CRC patients, especially in those at high risk or with p53-compromised tumors. Supplementation with sodium butyrate may also function as an adjunct to conventional chemotherapeutics—as supported by its suppressive action on *MCL-1* and other anti-apoptotic markers. Moreover, the dose-dependent effects on key gene networks provide a solid base for future efforts focused on developing controlled-release formulations or combination protocols to maintain effective butyrate levels in the tumor microenvironment.

Finally, several important limitations need to be considered. First, a single cell line was used in this study to minimize potential variability arising from using multiple cell lines. Indeed, the HCT-116 cell line is a widely recognized in vitro model for CRC due to its ease of culture, well-characterized genetic profile, homogeneity, tumorigenic capability, and high amenability to transfection [58,59]. However, it exhibits several important drawbacks, such as a lack of genetic/phenotypic diversity, specific drug resistance mechanisms, or in vivo modeling challenges due to the absence of a microenvironment and failure to account for immune–tumor interactions [58,59,60]. These aspects limit the generalizability of our results across the diverse spectrum of CRC types. Future research should, therefore, incorporate multiple cell lines to validate and extend these results.

Second, this was an in vitro study with a 24 h treatment duration. This relatively short duration limits the ability to observe long-term NaB effects (e.g., cumulative apoptosis, effect on cell cycle progression/resistance mechanisms), as well as the corresponding secondary outcomes (e.g., epigenetic changes, immune modulation, alterations in cell cycle progression). This timeframe may also fail to capture delayed or secondary effects of NaB treatment, which are crucial for understanding its full therapeutic potential. Despite these limitations, the present study, however, provides significant translational value by highlighting underexplored molecular targets (e.g., *MCL*-1, *PCNA*) and the dose-dependent effect of NaB, which are critical for optimizing therapeutic strategies. Moreover, the use of HCT-116 cells allowed us to conduct highly controlled analyses of gene expression and morphological changes, which are often challenging to isolate in vivo due to complexity and variability. These in vitro results serve as a necessary precursor for in vivo validation. Future studies should integrate animal models to evaluate NaB’s effects within the tumor microenvironment and assess its systemic bioavailability, pharmacodynamics, and potential immunomodulatory effects.

Third, the current study does not address the potential off-target effects of NaB on non-cancerous cells or other cellular pathways beyond those investigated. Nonetheless, it prioritizes understanding NaB’s mechanisms of action in cancer cells, with the observed dose-dependent effects providing critical insights into its therapeutic potential in CRC. The findings also provide a strong basis for future research to assess the selectivity of sodium butyrate and potential off-target effects, including studies in normal cell lines or co-culture systems that mimic healthy tissues.

Future research should expand to include other CRC cell lines, such as SW480 or LoVo, to capture heterogeneity in genetic mutations and therapeutic responses. Incorporating animal models will also allow us to investigate the systemic effects of sodium butyrate, including its interactions with the tumor microenvironment, bioavailability, and pharmacodynamics. Moreover, utilizing patient-derived CRC organoids could bridge the gap between in vitro findings and clinical applicability, enhancing the translational potential of our research.

## 5. Conclusions

Sodium butyrate exerts dose-dependent effects on HCT-116 colorectal cancer cells, significantly reducing viability, cell proliferation, and confluence at concentrations starting from 5 mM onward. It induces apoptosis and cell cycle arrest via upregulation of pro-apoptotic genes, such as *BAX*, *CASP3*, and *PUMA*, and downregulation of anti-apoptotic and proliferation markers, such as *BCL-2*, *MCL-1*, *PCN*A, and *Ki-67*. The expression of the tumor suppressor gene *TP53* was minimally affected, indicating that NaB acts largely through downstream mechanisms independent of TP53 functionality. Higher doses of NaB (>10 mM) result in pronounced cytotoxic effects, including morphological changes, nuclear fragmentation, and significant gene expression modulation. These findings highlight the potential of NaB as a therapeutic agent for colorectal cancer, particularly in tumors with compromised p53 functionality, and emphasize the need for further exploration of its mechanisms at higher concentrations.

## Figures and Tables

**Table 1:** Nucleotide sequences of primers for analyzed genes.

Gene	Forward	Reverse
*BAX*	TTTGCTTCAGGGTTTCATCC	AGACACTCGCTCAGCTTCTT
*BCL-2*	TTCTTTGAGTTCGGTGGGGT	GACTTCACTTGTGGCCCAG
*PCNA*	TTGATGAGGCGCGTGAATTT	GTTTGCAGCAGTGGGTGTG
*Ki-67* (*MKI67*)	ACCTACGGATTGAGACGTCAG	CTTCACTGTGTAGCCATTGTTGG
*CASP3*	GTGGAACTGACGATGATATGGC	CGCAAAGTGACTGGATGAACC
*PUMA* (*BBC3*)	CCTGGAGGGTCG0ACAGTACGA	GCCACCTTCCCATAGTTCCG
*TP53*	CACCATGAGCGCT0GCTCAGAT	TTGGGCAGTGCTCGCTTAGTG
*MCL-1*	AGGACACCCAGGAA0ACGAAG	GGTGCTGTTGACATCTAGGTTC
*NF-κB* (*NFKB1*)	TGCTTCTCTGACGTCT0GCTG	GCGTTGATGGTGGAGAGTGG
*CDKN1A* (*p21*)	CCTGGTGATGTCCGACCTG	CCATGAGCGCATCGCAATC

**Table 2:** Gene expression at different NaB concentrations.

Treatment Dose	BAX	BCL-2	PCNA	Ki-67	CASP3
5 mM	2.52 (0.42)	0.64 (0.10)	0.54 (0.08)	0.49 (0.12)	2.54 (0.48)
10 mM	9.37 (0.96) *	0.35 (0.05) ****	0.28 (0.004) ****	0.29 (0.05) *	9.16 (2.21)
15 mM	26.59 (3.14) ****	0.15 (0.02) ****	0.17 (0.070) ****	0.15 (0.04) ****	25.62 (1.08) **
20 mM	51.38 (3.65) ****	0.05 (0.02) ****	0.06 (0.01) ****	0.06 (0.03) ****	56.12 (9.75) ****
	**PUMA**	**TP53**	**MCL-1**	**NF-κ0B**	**CDKN1**
5 mM	2.22 (0.17)	0.73 (0.10)	0.90 (0.07)	0.63 (0.009)	0.60 (0.14)
10 mM	5.49 (0.72)	0.65 (0.06)	0.46 (0.07) ****	0.34 (0.05) ****	0.34 (0.05) *
15 mM	12.20 (3.15) *	0.50 (0.02) **	0.25 (0.03) ****	0.20 (0.03) ****	0.19 (0.02) ****
20 mM	34.59 (5.20) ****	0.38 (0.03) ****	0.13 (0.01) ****	0.08 (0.02) ****	00.13 (0.03) ****

Data are presented as mean values with one standard deviation (in parentheses). Marked values (*) indicate significant differences compared to the lowest treatment dose. (Dunnet’s tests, ****—*p* < 0.0001, ***— *p* < 0.001, **— *p* < 0.01, *— *p* < 0.05).

## Data Availability

All the data generated or analyzed during this study are included in this published article.
